# Comparative Gut Microbiome Differences between High and Low Aortic Arch Calcification Score in Patients with Chronic Diseases

**DOI:** 10.3390/ijms24065673

**Published:** 2023-03-16

**Authors:** Yi-Hsueh Liu, Po Peng, Wei-Chun Hung, Ping-Hsun Wu, Cheng-Yuan Kao, Pei-Yu Wu, Jiun-Chi Huang, Chih-Hsing Hung, Ho-Ming Su, Szu-Chia Chen, Chao-Hung Kuo

**Affiliations:** 1 Graduate Institute of Clinical Medicine, College of Medicine, Kaohsiung Medical University, Kaohsiung 807, Taiwan; 2 Department of Internal Medicine, Kaohsiung Municipal Siaogang Hospital, Kaohsiung 812, Taiwan; 3 Division of Cardiology, Department of Internal Medicine, Kaohsiung Medical University Hospital, Kaohsiung 812, Taiwan; 4 Welgene Biotech Co., Ltd., Taipei 115, Taiwan; 5 Department of Microbiology and Immunology, College of Medicine, Kaohsiung Medical University, Kaohsiung 807, Taiwan; 6 Division of Nephrology, Department of Internal Medicine, Kaohsiung Medical University Hospital, Kaohsiung 807, Taiwan; 7 Faculty of Medicine, College of Medicine, Kaohsiung Medical University, Kaohsiung 807, Taiwan; 8 Immunology Research Center, National Health Research Institutes, Zhunan, Miaoli 350, Taiwan; 9 Research Center for Precision Environmental Medicine, Kaohsiung Medical University, Kaohsiung 807, Taiwan; 10 Department of Pediatrics, Kaohsiung Medical University Hospital, Kaohsiung Medical University, Kaohsiung 807, Taiwan; 11 Department of Pediatrics, Kaohsiung Municipal Siaogang Hospital, Kaohsiung Medical University, Kaohsiung 812, Taiwan; 12 Division of Gastroenterology, Department of Internal Medicine, Kaohsiung Medical University Hospital, Kaohsiung Medical University, Kaohsiung 807, Taiwan

**Keywords:** microbiome, atherosclerosis, aortic arch calcification, microbial dysbiosis, chronic disease

## Abstract

Gut dysbiosis can induce chronic inflammation and contribute to atherosclerosis and vascular calcification. The aortic arch calcification (AoAC) score is a simple, noninvasive, and semiquantitative assessment tool to evaluate vascular calcification on chest radiographs. Few studies have discussed the relationship between gut microbiota and AoAC. Therefore, this study aimed to compare the microbiota composition between patients with chronic diseases and high or low AoAC scores. A total of 186 patients (118 males and 68 females) with chronic diseases, including diabetes mellitus (80.6%), hypertension (75.3%), and chronic kidney disease (48.9%), were enrolled. Gut microbiota in fecal samples were analyzed by sequencing of the 16S rRNA gene, and differences in microbial function were examined. The patients were divided into three groups according to AoAC score, including 103 patients in the low AoAC group (AoAC ≤ 3), 40 patients in the medium AoAC group (3 < AoAC ≤ 6), and 43 patients in the high AoAC group (AoAC > 6). Compared to the low AoAC group, the high AoAC group had a significantly lower microbial species diversity (Chao1 index and Shannon index) and increased microbial dysbiosis index. Beta diversity showed that the microbial community composition was significantly different among the three groups (*p* = 0.041, weighted UniFrac PCoA). A distinct microbial community structure was found in the patients with a low AoAC, with an increased abundance at the genus level of *Agathobacter*, *Eubacterium coprostanoligenes* group, Ruminococcaceae UCG-002, *Barnesiella*, *Butyricimonas*, *Oscillibacter*, Ruminococcaceae DTU089, and *Oxalobacter*. In addition, there was an increased relative abundance of class Bacilli in the high AoAC group. Our findings support the association between gut dysbiosis and the severity of AoAC in patients with chronic diseases.

## 1. Introduction

Atherosclerosis is a systemic process involving lipid accumulation and inflammation of arterial beds and thickening of arterial walls. It is a major cause of cardiovascular disease (CVD), which has been a leading cause of death worldwide for many years [1]. Vascular calcification is a complication of advanced atherosclerosis, and it can be detected using imaging techniques such as computed tomography (CT), radiography, echocardiography, and vascular ultrasound [2,3]. Among them, CT is still considered the gold standard for quantifying coronary or aortic calcification [4,5]. However, CT is expensive and exposes the patients to high doses of radiation and the risk of cancer [6]. The aortic arch calcification (AoAC) score is a simple, noninvasive, semiquantitative assessment tool used to evaluate vascular calcification on chest radiographs [7]. The AoAC score has also been highly correlated with the AoAC volume as determined by multidetector CT [7]. AoAC detectable on chest X-rays has been strongly associated with coronary artery calcification [8] and cardiovascular events [9], and also with the severity of coronary artery disease as evaluated by the SYNTAX score in patients with acute coronary syndrome [10]. Previous studies have demonstrated that AoAC could predict renal function progression in chronic kidney disease (CKD) patients [11,12], and be independently associated with cardiovascular and all-cause mortality in both CKD and end-stage renal disease patients [12,13,14,15]. In addition, AoAC assessed by chest X-ray has been shown to be an independent risk factor for all-cause mortality and CVD in the general population, with a dose–response relationship in a large cohort study with 27,166 Chinese participants aged ≥ 50 years and free of CVD [16]. Taken together, AoAC is an effective modality to detect the severity of vascular calcification, and it plays an important role as a risk factor and prognostic marker in older populations and in those with chronic diseases.

The gut microbiota comprises trillions of microbes, including bacteria, archaea and viruses, that colonize the entire gut [17]. The microbiota is a virtual organ in humans that moderates homeostasis and disease. In a healthy host, these effects are mostly symbiotic and influence immunity, nutrition, metabolism, and energy [18]. Changes in the composition of the gut microbiota, called gut dysbiosis, have been associated with the development of atherosclerosis-related CVD via various pathways [19].

The development of modern molecular techniques has allowed taxonomically heterogeneous microbial communities to be examined more comprehensively. The importance of some species in CVD has also been demonstrated [20]. Even though associations of specific bacterial taxa with atherosclerosis have been shown, many questions remain with regards to how the microbiome contributes to atherosclerosis [21]. Previous studies have demonstrated associations between changes in gut microbiota composition with coronary artery disease, carotid artery disease, arteriosclerotic plaque, and pulse wave velocity [20,21,22,23,24,25,26,27,28]. However, no studies have discussed the relationship between the gut microbiota and AoAC. Therefore, this study aimed to compare the intestinal microbiome composition between patients with chronic diseases and high or low AoAC scores.

## 2. Results

### 2.1. Patient Characteristics

A total of 186 individuals (118 males and 68 females), where the average age was 65.7 years, with chronic disease, including diabetes mellitus (80.6%), hypertension (75.3%), and chronic kidney disease (48.9%), were enrolled in the study. The patients were divided into three groups according to AoAC score, including 103 patients in the low AoAC group (AoAC score ≤ 3), 40 patients in the medium AoAC group (3 < AoAC score ≤ 6), and 43 patients in the high AoAC group (AoAC score > 6), were enrolled in this study. The baseline characteristics of the enrolled patients with chronic disease are reported in Table 1. The mean age was 61.6 years in the low AoAC group and 71.6 years in the high AoAC group. Compared to the low AoAC group, the high AoAC group had an older age, higher diastolic blood pressure, higher hypertension prevalence with more Beta-blocker and calcium channel blocker medication use, lower hemoglobin level, and lower eGFR. There was no significant difference between the high and low groups in sex, BMI, systolic blood pressure, diabetes mellitus, CKD, CVD, cerebrovascular disease, and other medication use (including angiotensin-converting enzyme inhibitor/angiotensin receptor blocker, diuretic, statins, fibrate, oral hypoglycemic agents and insulin). For clinical laboratory data, the high and low AoAC groups had no significant difference between albumin, HbA1c, triglycerides, total cholesterol, HDL-cholesterol, LDL-cholesterol, total calcium and phosphorous level.

### 2.2. Gut Microbiota Profile Differs among Different AoAC Severity

**Figure 1:**
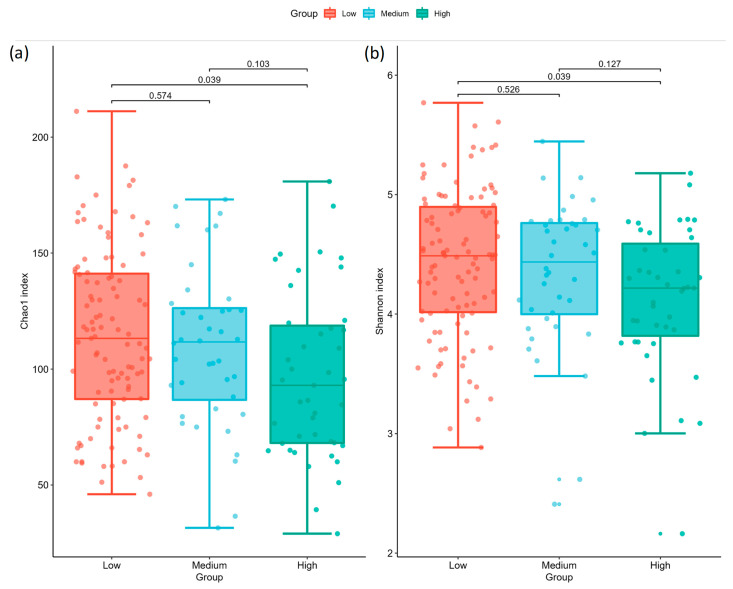
Comparisons of alpha diversity indices for (**a**) Chao1 and (**b**) Shannon across AoAc levels. Each dot represents a diversity index of one sample. The center lines in boxes represent the medians; box limits indicate the 25th and 75th percentiles; whiskers extend 1.5 times the interquartile range from the 25th and 75th percentiles. The *p* values for pairwise comparisons are shown on each pair of brackets.

**Figure 2:**
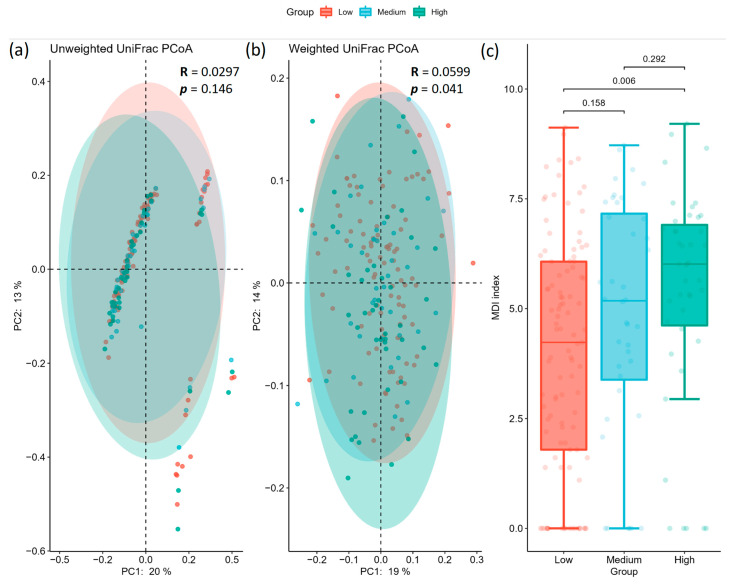
Comparisons of the microbiota β-diversity and MDI index. PCoA representation using (**a**) unweighted and (**b**) weighted normalized UniFrac distance matrix, while each dot represents one sample. (**c**) MDI index comparison where *p* values for pairwise comparisons are shown on each pair of brackets.

The alpha diversity analysis showed that microbial community richness differed among groups (Kruskal–Wallis test for all groups using Chao1 index: H = 6.577, *p* = 0.037; Kruskal–Wallis test for all groups using Shannon index: H = 6.497, *p* = 0.038). Pairwise results are summarized in Figure 1 and Table 2 (alpha diversity), where the alpha diversity indices were significantly lower in the high AoAC group (Chao1 index: 96.89 ± 36.168; Shannon’s index: 4.133 ± 0.601) than that at the low AoAC group (Chao1 index: 114.313 ± 36.902; Shannon’s index: 4.428 ± 0.617), with no significant difference between the low AoAC and medium AoAC groups (Chao1 index: 109.361 ± 34.525; Shannon’s index: 4.336 ± 0.626). The beta diversity analysis showed that the microbial community composition was significantly different among the groups (ANOSIM for overall significance using unweighted UniFrac: R = 0.0297, *p* = 0.146; ANOSIM for overall significance using weighted normalized UniFrac: R = 0.0599, *p* = 0.041). Specifically, while the results of pairwise ANOSIM tests using unweighted UniFrac matrices did not reach significance (Figure 2a, Table 3a), adopting weighted normalized UniFrac dissimilarities revealed the distinct clustering patterns of the low AoAC and high AoAC groups (Figure 2b, Table 3b). In addition, the high AoAC group had higher MDI than the low AoAC group (Figure 2c).

### 2.3. Specific Microbial Taxa Are Associated with Different AoAC Severity

**Figure 3:**
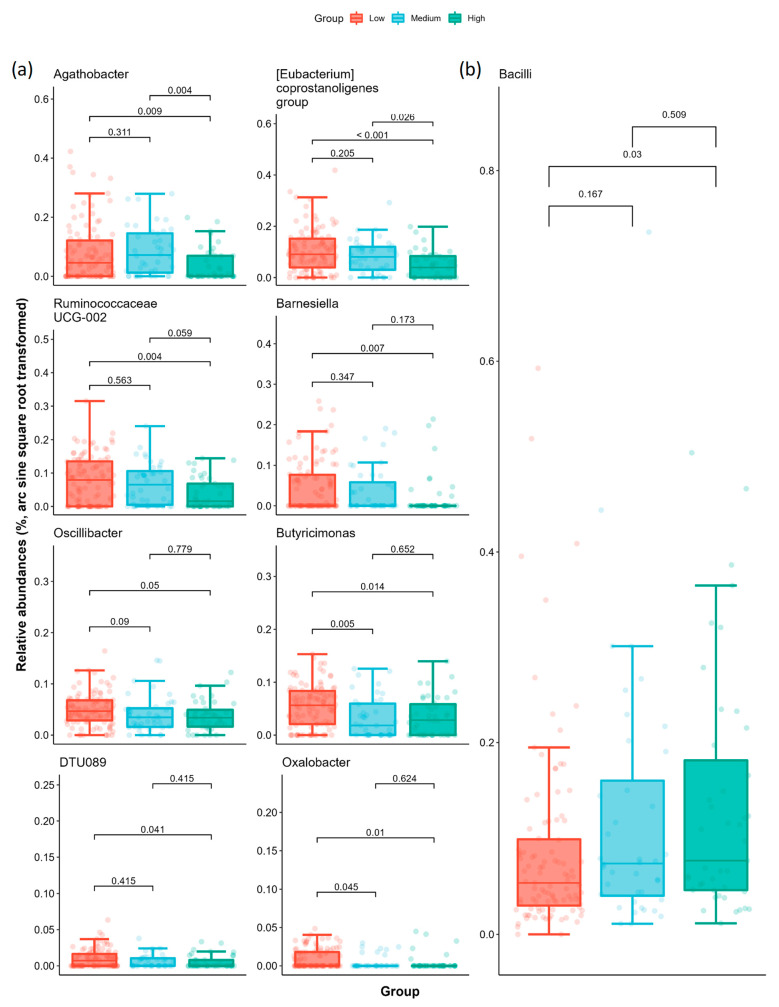
Relative abundances of significantly differed taxa among low, medium, and high AoAc groups: (**a**) genus and (**b**) class level. The center lines in boxes represent the medians; box limits indicate the 25th and 75th percentiles; and whiskers extend 1.5 times the interquartile range from the 25th and 75th percentiles. The *p* values for pairwise comparisons using pairwise Dunn’s test with Holm’s correction are shown on each pair of brackets.

We analyzed the relative abundance of taxa to identify the taxa differences between AoAC severities using LEfSe (Linear discriminant analysis Effect Size) to discriminate features associated with AoAc levels (Figure S1a,b). Based on the logarithmic LDA (Linear discriminant analysis) score > 2 and Kruskal–Wallis test *p* < 0.05, we identified 47 taxa among the three groups at different taxonomic levels, including 15 genera and 10 species. At the genus level, the low AoAC group presented an increased relative abundance of *Agathobacter*, *Eubacterium coprostanoligenes* group, Ruminococcaceae UCG-002, *Barnesiella*, *Butyricimonas*, *Oscillibacter*, Ruminococcaceae DTU089, and *Oxalobacter* (Figure 3). As for the high AoAC group, we found an increased relative abundance of class Bacilli. MaAsLin2 results reconciled the consistency in those biomarkers at genus level after adjusting for the covariates (age, sex, HTN, and CKD), whereas it failed to detect the significance at class level (the statistical results are presented in supplementary information Table S1 and Figure S2). Further analysis of the KEGG module also revealed a difference in gut microbiota pathway in low AoAC group compared with high AoAC group (Figure S3).

## 3. Discussion

The aim of this study was to investigate the relationship between microbiota composition and AoAC in patients with chronic diseases. We found structural differences in the stool microbial communities between the high and low AoAC groups, with reduced alpha diversity (Chao1 index and Shannon index) and increased MDI in the high AoAC group. Beta diversity analysis showed that the microbial community composition was significantly different among the three different AoAC severity groups (*p* = 0.041, weighted UniFrac PCoA). A distinct microbial community structure was found in the patients with a low AoAC score, with increased abundances at the genus level of *Agathobacter*, *Eubacterium coprostanoligenes* group, Ruminococcaceae UCG-002, *Barnesiella*, *Butyricimonas*, *Oscillibacter*, Ruminococcaceae DTU089, and *Oxalobacter*. In addition, there was an increased relative abundance of class Bacilli in the high AoAC group.

The microbiota may influence atherosclerosis by promoting plaque development via direct and distant infection, activation of the immune system, alteration of cholesterol metabolism, and production of bacterial metabolites [29]. Emerging data have demonstrated strong associations between the gut microbiota and risk factors for the development of CVD, including atherosclerosis, inflammation, obesity, insulin resistance, platelet hyperactivity, and plasma lipid abnormalities [30,31,32]. Several studies of humans and animal models have demonstrated associations between gut microbial metabolites such as trimethylamine-N-oxide (TMAO), short-chain fatty acids (SCFAs), and bile acid metabolites (amino acid breakdown products) with CVD [30,33,34]. TMAO has been shown to increase the risk of CVD by altering cholesterol and bile acid metabolism, activating inflammatory pathways, and promoting foam cell formation and platelet hyperactivation, whereas SCFAs have been shown to contribute to atherosclerosis and hypertension by different mechanisms [32,35]. Several studies have reported the presence of genetic material from a wide range of bacteria in atherosclerotic plaque [23,24,25,26]. In a study conducted in Moscow, enterotyping identified two clusters according to the alpha diversity, and the cluster with lower diversity was associated with higher intima-media thickness [27]. In addition, in a Chinese study, the severity of coronary artery disease was significantly associated with changes in the composition of both gut microbiota and metabolites, represented by *Roseburia*, *Klebsiella*, Clostridium IV and Ruminococcaceae [22]. In a Swedish study, greater enrichment of the genus *Collinsella* was found in patients with carotid artery stenotic atherosclerotic plaque, whereas in healthy controls, greater enrichment of *Roseburia* and *Eubacterium* was found [21]. In a study of the TwinsUK cohort, the association of carotid–femoral pulse wave velocity (PWV) and gut microbiome composition was studied in 617 middle-aged women, and the results showed that PWV was negatively correlated with gut microbiome alpha diversity after adjusting for covariates [28]. Growing evidence suggests that higher alpha diversity, a measure of bacterial richness or evenness, is linked to a better health status and temporal stability of the gut microbiome [36], whereas a relative lack of alpha diversity is linked to poor health. Reduced gut microbial alpha diversity has been found in individuals with a variety of chronic illnesses, including obesity, inflammatory bowel disease, hypertension, and type 2 diabetes, as well as older subjects with frailty [37,38,39,40,41]. In our study, the high AoAC group had lower alpha diversity and a higher MDI than the low AoAC group, and there was a significant difference in alpha diversity between the two groups. Thus, our findings demonstrate that the degree of AoAC detectable on chest X-rays is associated with the alpha diversity and composition of the gut microbiota.

We also found that several butyrate-producing bacteria were markedly increased in the low AoAC group, including genus *Agathobacter*, Ruminococcaceae UCG-002, Ruminococcaceae DTU089, *Oscillibacter* and *Butyricimonas*. Butyrate is a main SCFA and a primary colonocyte energy source, and it also maintains the integrity of the gut barrier and homeostasis of the intestine by anti-inflammatory processes [42,43,44]. Several studies have reported an association between butyrate and reduced appetite, potentially due to increases in glucagon-like peptide-1 (GLP-1), leading to reduced energy intake and a decrease in body weight [45,46,47]. Moreover, the administration of butyrate has been shown to improve metabolic disorders, including hepatic lipogenesis and hyperglycemia through glucose transporter type 4, GLP-1, and AMP-activated protein kinase [48,49]. Our results support a link between specific butyrate-producing bacteria and low AoAC in patients with chronic diseases.

Bacteria have been associated with many chronic diseases in previous studies, and they may play a specific role in reducing AoAC. *Agathobacter* is a member of the Lachnospiraceae family, which can produce butyrate through dietary fiber fermentation [50]. *Agathobacter* has been reported to be more prevalent in normoglycemic subjects than in those with prediabetes [51]. Ruminococcaceae UCG-002, Ruminococcaceae DTU089, and *Oscillibacter* all belong to the Ruminococcaceae family. Ruminococcaceae are less abundant in hypertensive patients than in normotensive patients [52], and they were negatively correlated with PWV in the TwinsUK cohort [28]. Ruminococcaceae have been negatively correlated with metabolic disease in humans [53]. Jiant et al. demonstrated an association between Ruminococcaceae UCG-002 with a lower risk of metabolic syndrome, type 2 diabetes mellitus, and dyslipidemia, and also a significantly inverse association with BMI [54]. Ruminococcaceae DTU089 have been shown to be more abundant in subjects with lower protein intake [55]. *Oscillibacter* have been shown to be significantly enriched in healthy subjects, and to be reduced in patients with non-alcoholic fatty liver disease [56] and hypertension [57]. A Mendelian randomization analysis demonstrated that *Oscillibacter* had a causal effect on reducing blood triglyceride concentrations, and lowering BMI and waist-hip ratio [58]. Previous studies have reported decreased abundances of the Lachnospiraceae and Ruminococcaceae families in patients with nonalcoholic steatohepatitis [56,59] and coronary artery disease [22]. *Butyricimonas* is a Gram-negative anaerobic bacterial genus of the family Odoribacteraceae. Treatment of metabolic disorders with metformin and statins has been shown to significantly increase the relative abundance of *Butyricimonas* in the gut, and this has been significantly associated with metabolic parameters [60,61]. Lee et al. reported that *Butyricimonas* could prevent high fat diet-induced diabetes and metabolic disorders in mice via the GLP-1 receptor [62]. *Butyricimonas* has also been negatively correlated with insulin resistance and obesity in humans [63,64].

We also found that some non-butyrate-producing bacteria may be involved in certain metabolic pathways that are associated with a low AoAC. The *Eubacterium coprostanoligenes* group play a major role in the metabolism of coprostanol, leading to a hypocholesterolemic effect [65,66]. Greater cholesterol absorption has been reported in the gut of patients with atherosclerotic CVD [67,68]. Alterations in the gut microbial community have been directly related to the rate of cholesterol conversion to coprostanol, and highly efficient cholesterol transformation to coprostanol has been associated with a lower risk of CVD [67,69]. Karlsson et al. reported that *Eubacterium* were enriched in healthy controls, and significantly negatively correlated with known risk factors for atherosclerosis including white blood cell count, low-density lipoprotein, and cholesterol in patients with atherosclerosis [21]. *Barnesiella* have been shown to induce the production of SCFAs in the gut and to indirectly or directly have an anti-inflammatory effect through an increase in SCFAs in the intestinal tract [70]. *Barnesiella* have also been associated with the fermentation of carbohydrates, pathogenic bacteria inhibition, and regulation of immunity [70,71,72]. *Oxalobacter* have been shown to play a key role in the degradation of oxalate in the intestinal tract, and to potentially have the ability to reduce the risk of calcium oxalate kidney stones [73,74]. Oxalate induces reactive oxygen species, which promote inflammation and cause systemic oxidation and vascular endothelial cell injury [75,76]. Li et al. reported that calcium oxalate calculi were significantly associated with abdominal aortic calcification after adjusting for confounding factors related to vascular calcification [74]. However, further studies are needed to investigate the specific pathophysiological mechanisms between oxalate and aortic calcification.

Several limitations should be mentioned. First, a cross-sectional study can only provide data of relative bacteria abundance at a single point in time, and therefore we could not make causal inferences. Further follow-up studies are needed to confirm our results. Second, we used fecal samples to assess the microbiota, and this may differ from the microbiota from other parts of the intestine. In addition, 16s rRNA sequencing is limited as it cannot differentiate viable from unviable bacteria. Therefore, a significant portion of the taxa identified by sequencing may not be metabolically active. Third, several butyrate-producing microbes were associated with low AoAC in our study; however, butyric acid was not investigated in this study. Fourth, as healthy individuals were not included in our study, a comparison between the control group and participants with chronic disease was not feasible. We will include more healthy individuals as the control group and other chronic disease patients in the future to analyze the role of gut microbiota in vascular calcification with a larger dataset. Fifth, the high AoAC group was older and had a higher proportion of hypertension and CKD than the low AoAC group, which may have affected the distribution of AOAC severity, although we had used MAsLin2, where age, sex, hypertension, and CKD were included as random effects to adjust for possible confounding impacts. Finally, the study was performed in Asian patients with chronic diseases, and they may have had a different diet compared to other populations. Therefore, the effect of diet on the gut microbiome should be interpreted with caution.

In conclusion, our findings support the association between gut dysbiosis and the severity of AoAC in patients with chronic diseases. A distinct microbial community structure and taxa features were found in the patients with a low AoAC score. However, it is still not clear how these particular gut bacteria prevent vascular calcification. Further in-depth studies are needed to investigate the relationship between these specific bacteria and AoAC.

## 4. Materials and Methods

### 4.1. Study Participants

From March 2020 through July 2020, 186 participants (118 males and 68 females) with chronic diseases were recruited from the outpatient department of Kaohsiung Municipal Siaogang Hospital. In this study, chronic diseases were defined as hypertension, diabetes or CKD that had been treated for more than 3 months. Eligible participants were aged between 35 and 99 years. Patients with active malignancies, abdominal cancer, radiation therapy to the abdomen, acute or chronic inflammation, and those who were prescribed antibiotics within 3 months before enrollment were excluded. The Institutional Review Board of Kaohsiung Medical University Hospital approved this study (KMUHIRB- G(II)-20190014; 28 May 2019), and all participants provided written informed consent. All methods were performed in accordance with relevant guidelines.

### 4.2. Demographic, Medical, and Laboratory Data

Demographic data, age, sex, body mass index (BMI), medical history, medications, and biochemical data were obtained from electronic medical records. Hypertension was defined as systolic blood pressure/diastolic blood pressure ≥140/85 mmHg or the use of antihypertensive drugs. Diabetes was defined as a glycated hemoglobin (HbA1c) level ≥6.5% or the use of antidiabetic agents. CKD was defined as kidney damage or estimated glomerular filtration rate (eGFR) <60 mL/min/1.73 m^2^ for ≥3 months. Overnight fasting blood samples were obtained for biochemical data including hemoglobin, albumin, fasting glucose, HbA1c, eGFR (calculated using the 4-variable Modification of Diet in Renal Disease study equation [77]), triglycerides, total cholesterol, high-density lipoprotein (HDL) cholesterol, low-density lipoprotein (LDL) cholesterol, uric acid, total calcium and phosphorous.

### 4.3. Evaluation of AoAC by Chest Radiography

A single experienced radiologist who was blinded to the clinical information of the patients reviewed all chest radiographs. Calcification of the aortic arch was assessed by dividing the aortic arch on chest radiographs into 16 sections according to their circumference [7]. The AoAC score was determined as the number of sections with vascular calcification.

### 4.4. Fecal Sample Collection and Bacterial 16S rRNA Amplicon Sequencing and Processing

Each patient received a small cylindrical receptacle enclosed in a zippered bag and wrapped in a small brown paper bag. The receptacle was hermetically sealed and sterile. The receptacles were used by the patients to collect their own feces, which were then frozen at home before being brought to the hospital. Fecal samples were collected on the same day as or the night before biochemical data were collected. The fecal samples were sent to the laboratory immediately at room temperature. Prior to deoxyribonucleic acid (DNA) extraction, the samples were frozen in a laboratory freezer at −20 °C.

Bacterial genomic DNA from the samples was extracted using a QIAamp PowerFecal DNA Kit (Qiagen), with the concentration adjusted to 5 ng/ul. The16S rRNA gene V3-V4 region was amplified by specific primers (341F: 5′-CCTACGGGNGGCWGCAG-3′, 806R: 5′- GACTACHVGGGTAT CTAATCC -3′) and sequenced on an Illumina MiSeq platform for paired 300-bp reads.

Paired-end raw FASTQ reads were processed using Trimmomatic [78], in which adapter trimming and low-quality (average QV < 20) read filtering were performed. Subsequently, primer removal was achieved using Cutadapt [79], in which the minimum length was set to 150 kb. The DADA2 [80] pipeline was used for error modelling and ASV (amplicon sequence variant) construction per sample, in which read pairs were merged with ≥20 bp overlap (allowing for 0 mismatches), resulting in 868 ASVs with a total frequency of 10,413,122 and an average of 55,984.527 ± 8090.5082 per sample.

### 4.5. Statistical and Bioinformatics Analyses of the Microbiota

As uneven sampling depth may increase the probability of false conclusions [81], we rarified the ASV dataset to 27,919 reads per sample (the minimum library size among all samples) and then used it for alpha and beta diversity estimation. For alpha diversity, we calculated the Chao1 [82] and Shannon [83] indices. Pairwise Kruskal–Wallis tests, where *p* values were corrected following the Benjamini–Hochberg procedure [84], were used to compare the alpha diversity indices among groups. Between-group (beta) diversity was assessed with principal coordinate analysis (PCoA) using unweighted and weighted normalized UniFrac [85] distance matrices. To investigate the impact of AoAC on microbial community composition, we used pairwise ANOSIM (analysis of similarity) tests [86], with 999 permutations applied to unweighted and weighted normalized UniFrac dissimilarity measures, followed by Benjamini–Hochberg correction [84]. Additionally, the microbial dysbiosis index (MDI) [87] was calculated to assess the association between the AoAC level and degree of dysbiosis. Differences among groups were examined using the non-parametric Kruskal–Wallis test and post-hoc Dunn’s all-pairs comparison test.

Taxonomy classification was performed using the q2-feature-classifier plugin in QIIME 2 [88], with a confidence threshold of 0.7 (default) to search the SILVA reference database (release v132, L7 taxonomy), resulting in 111 taxa. To identify the potential microbial biomarkers associated with AoAC, we analyzed relative abundances of taxa using LEfSe (linear discriminant analysis effect size) [89], where logarithmic LDA scores for discriminative features and the significance level for the on-parametric factorial Kruskal–Wallis test were set to 2 and 0.05, respectively. In addition, significant taxa identified by LEfSe were tested using pairwise Dunn’s test with Holm’s procedure [90], to examine the association between a given biomarker and AoAc level.

To validate the findings of LEfSe analysis, we further compared abundances at the genus level between low and high AoAC groups using Multivariate Association with Linear Models 2 (MaAsLin2) [91], where age, sex, hypertension, and CKD were included as random effects to adjust for possible confounding impacts. With arcsine square-root transformation, taxa with relative abundances of at least 20% of all samples were tested.

### 4.6. Functional Annotation

To predict functional profiling of the microbiota, Phylogenetic Investigation of Communities by Reconstruction of Unobserved States version 2 (PICRUSt2) [92] was used to estimate KEGG [93] pathway abundances across samples. The significance of functional differences inferred from sequence variants was analyzed using the Kruskal–Wallis test followed by Dunn’s all-pairs comparison test.

## Figures and Tables

**Table 1:** Clinical characteristics of the study population.

Characteristics	Low AoAC Group AoAC ≤ 3 (N = 103)	Medium AoAC Group 3 < AoAC ≤ 6 (N = 40)	High AoAC Group AoAC > 6 (N = 43)	*p*
AoAC score	1.17 ± 1.20	4.75 ± 0.58 *	8.51 ± 2.06 *^†^	< 0.001
Age (year)	61.56 ± 9.54	69.93 ± 7.98 *	71.63 ± 9.31 *^†^	< 0.001
Male (%)	66.99%	67.50%	51.16%	0.164
BMI	26.65 ± 4.83	26.38 ± 4.12	26.13 ± 3.22	0.802
SBP (mmHg)	134.91 ± 17.30	137.03 ± 17.55	141.30 ± 21.51	0.163
DBP (mmHg)	73.55 ± 12.98	68.53 ± 9.58	67.33 ± 12.37 *	0.007
**Comorbidities**				
Diabetes mellitus	85.44%	65.00% *	83.72%	0.017
Hypertension	68.93%	75.00%	90.70% *	0.021
Chronic kidney disease	40.78%	57.50%	60.47%	0.045
Cardiovascular disease	6.80%	15.00%	9.30%	0.314
Cerebrovascular disease	0.97%	5%	2.33%	0.332
**Medications**				
ACEI/ARB	66.02%	62.50%	67.44%	0.887
Beta-blocker	14.56%	25.00%	34.88% *	0.020
CCB	44.66%	45.00%	69.77% *	0.016
Diuretic	21.36%	30.00%	30.23%	0.397
Statins	66.02%	67.50%	72.09%	0.777
Fibrate	4.85%	10.00%	0%	0.106
OHA	78.64%	62.50%	81.40%	0.081
Insulin	16.50%	17.50%	16.28%	0.987
**Clinical laboratory data**				
Hemoglobin (g/dL)	13.48 ± 1.92	12.27 ± 2.20 *	12.46 ± 2.24 *	0.001
Albumin (g/dL)	4.17 ± 0.24	4.12 ± 0.23	4.11 ± 0.24	0.269
HbA1c (%)	7.18 ± 1.22	6.58 ± 1.02 *	6.99 ± 1.10	0.020
eGFR (mL/min/1.73 m^2^)	56.19 ± 22.55	43.54 ± 24.72 *	44.50 ± 22.02 *	0.002
Triglycerides (mg/dL)	122.42 (108.07–136.77)	143.25 (113.93–172.57)	131.98 (104.60–159.35)	0.377
Total cholesterol (mg/dL)	162.46 ± 33.74	164.80 ± 44.80	165.42 ± 34.79	0.884
HDL-cholesterol (mg/dL)	46.58 ± 10.12	43.26 ± 9.56	48.53 ± 11.45	0.064
LDL-cholesterol (mg/dL)	86.07 ± 28.16	83.93 ± 34.51	81.37 ± 21.21	0.651
Total Calcium (mg/dL)	9.17 ± 0.37	9.07 ± 0.39	9.17 ± 0.47	0.359
Phosphorous (mg/dL)	3.51 ± 0.52	3.76 ± 0.81	3.68 ± 0.68	0.063

Data are shown as mean ± standard error of the mean. Abbreviations. AoAC, aortic arch calcification; BMI, body mass index; SBP, systolic blood pressure; DBP, diastolic blood pressure; ACEI, angiotensin-converting enzyme inhibitor; ARB, angiotensin receptor blocker; CCB, calcium channel blocker; OHA, oral hypoglycemic agent; eGFR, estimated glomerular filtration rate; HDL, high-density lipoprotein; LDL, low-density lipoprotein. * *p* < 0.05 compared with AoAC ≤ 3; ^†^ *p* < 0.05 compared with 3 < AoAC ≤ 6.

**Table 2:** Pairwise comparisons (Kruskal–Wallis test) of alpha diversity between samples using (**a**) Chao1 and (**b**) Shannon indices, where adjusted *p* was computed following the Benjamini–Hochberg procedure.

**(a) Chao1**				
Group 1	Group 2	H	*p*	Adjusted *p*
Low	Medium	0.316048	0.573992	0.573992
Medium	High	3.322329	0.068345	0.102517
Low	High	6.179246	0.012926	0.038777
**(b) Shannon**				
Group 1	Group 2	H	*p*	Adjusted *p*
Low	Medium	0.402124	0.525994	0.525994
Medium	High	2.966860	0.084987	0.127480
Low	High	6.189636	0.012850	0.038550

Low: Low AoAC Group, AoAC ≤ 3 (N = 103). Medium: Medium AoAC group, 3 < AoAC ≤ 6 (N = 40). High: High AoAC group, AoAC > 6 (N = 43).

**Table 3:** Pairwise comparisons of beta diversity between samples using (**a**) unweighted and (**b**) weighted normalized UniFrac distances, where adjusted *p*-value was computed following the Benjamini–Hochberg procedure.

**(a) unweighted**				
Group 1	Group 2	R	*p*	Adjusted *p*
Low	Medium	0.029929	0.232	0.278
Medium	High	0.004101	0.278	0.278
Low	High	0.034003	0.168	0.278
**(b) weighted**				
Group 1	Group 2	R	*p*	Adjusted *p*
Low	Medium	0.022670	0.283	0.4245
Medium	High	−0.003829	0.544	0.5440
Low	High	0.105367	0.006	0.0180

Low: Low AoAC Group, AoAC ≤ 3 (N = 103). Medium: Medium AoAC group, 3 < AoAC ≤ 6 (N = 40). High: High AoAC group, AoAC > 6 (N = 43).

## Data Availability

Data may be available upon request to interested researchers. Please send data requests to: Szu-Chia Chen. Division of Nephrology, Department of Internal Medicine, Kaohsiung Medical University Hospital, Kaohsiung Medical University.

## Supplementary Materials

The following are available online at https://www.mdpi.com/article/10.3390/ijms24065673/s1.
