# Supplementary material for: Comparative Gut Microbiome Differences between High and Low Aortic Arch Calcification Score in Patients with Chronic Diseases
Source: Int J Mol Sci. 2023 Mar 16;24(6):5673. doi: 10.3390/ijms24065673 (PMC10059004; doi:10.3390/ijms24065673)
Supplement: Supplementary file 1 [file ijms-24-05673-s001.zip › ijms-2226513-supplementary.pdf]

Figure S1. Taxonomic differences across groups with different AoAC levels identified by LEfSe analysis. (a) Histogram of LDA score comparison. (b) Circular cladogram reporting results for taxonimic ranks, in which each circle from the center to the periphery represent higher to lower taxonomic rank, with phylum (p__), class (c__), order (o__), family (f__) and genus (g__) denoted in the legend.

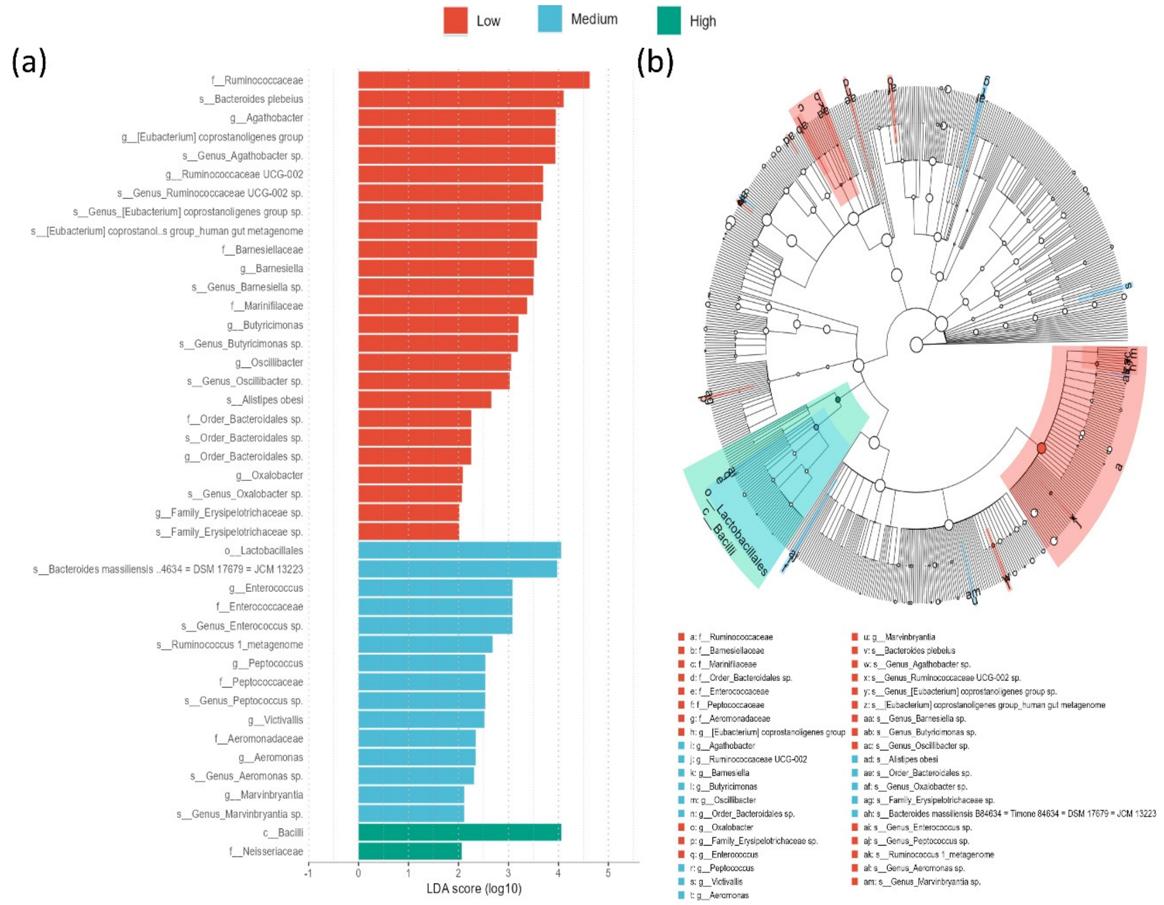

Figure S2. Relative abundances of significantly differed taxa among low and high AoAc groups.

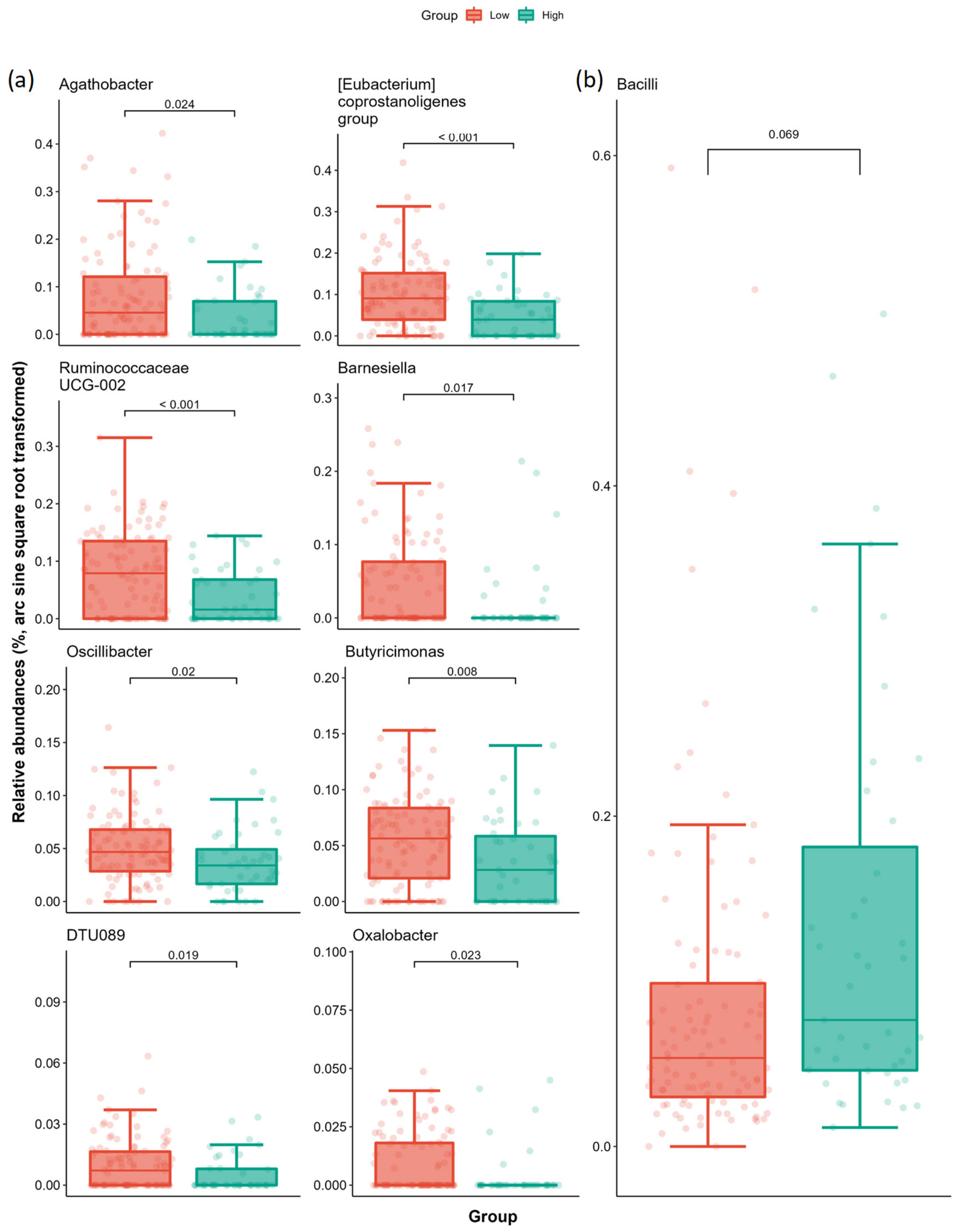

P-values calculated with MaAsLin2 after adjusting for age, sex, hypertension, and CKD: (a) genus and (b) class level. The center lines in boxes represent the medians; box limits indicate the 25th and 75th percentiles; whiskers extend 1.5 times the interquartile range from the 25th and 75th percentiles. P values are reported on each pair of brackets.

Figure S3. Relative abundances of functional metagenomic pathways predicted by PICRUSt2.

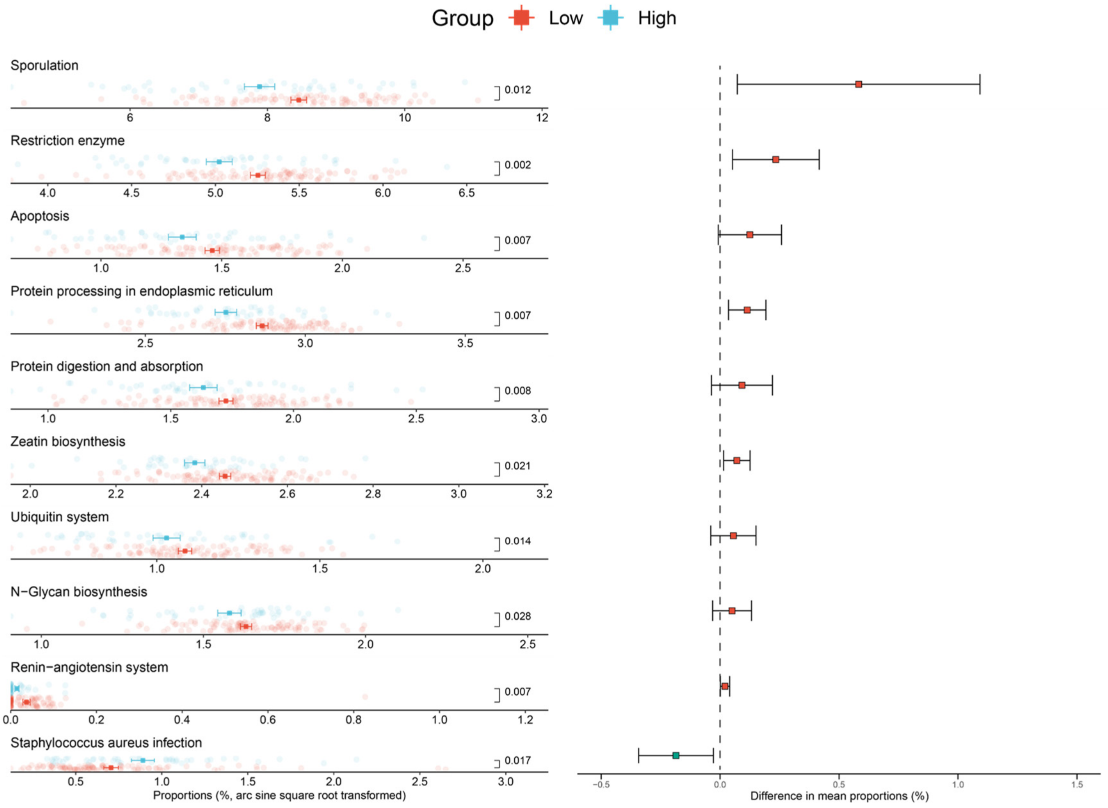

Squares and whiskers represent the mean and standard error, while each circle indicates the predicted pathway abundance of one sample

Table S1. Results from MaAsLin2 modelling adjusted for the covariates (age, sex, hypertention, and CKD).

Taxonomic Rank	Taxa	Coef[1]	Stderr[2]	N[3]	N not 0[4]	p-value
Genus	[Eubacterium] coprostanoligenes group	-0.057	0.014	146	117	0.000
	Ruminococcaceae UCG-002	-0.040	0.012	146	98	0.001
	Agathobacter	-0.038	0.016	146	86	0.024
	Barnesiella	-0.027	0.011	146	58	0.017
	Butyricimonas	-0.019	0.007	146	111	0.008
	DTU089	-0.005	0.002	146	66	0.019
	Oscillibacter	-0.014	0.006	146	132	0.020
	Oxalobacter	-0.005	0.002	146	47	0.023
Class	Bacilli	0.038	0.021	146	144	0.069

[1] The model coefficient value (effect size)
[2] The standard error from the model
[3] The total number of samples used in the model
[4] The total of number of these samples in which the feature is non-zero
